# Logistic regression technique is comparable to complex machine learning algorithms in predicting cognitive impairment related to post intensive care syndrome

**DOI:** 10.1038/s41598-023-28421-6

**Published:** 2023-02-11

**Authors:** TingTing Wu, YueQing Wei, JingBing Wu, BiLan Yi, Hong Li

**Affiliations:** 1 The School of Nursing, Fujian Medical University, Fujian, China; 2 Research Center for Nursing Theory and Practice, Fujian Provincial Hospital, No 134, East Street, Gulou District, Fuzhou, 35001 Fujian China; 3 Respiratory and Intensive Care Unit, Fujian Provincial Hospital, Fujian, China; 4 Medical Intensive Care Unit, Fujian Provincial Hospital, Fujian, China

**Keywords:** Medical research, Risk factors

## Abstract

To evaluate the performance of machine learning (ML) models and to compare it with logistic regression (LR) technique in predicting cognitive impairment related to post intensive care syndrome (PICS-CI). We conducted a prospective observational study of ICU patients at two tertiary hospitals. A cohort of 2079 patients was screened, and finally 481 patients were included. Seven different ML models were considered, decision tree (DT), random forest (RF), XGBoost, neural network (NN), naïve bayes (NB), and support vector machine (SVM), and compared with logistic regression (LR). Discriminative ability was evaluated by area under the receiver operating characteristic curve (AUC), calibration belt plots, and Hosmer–Lemeshow test was used to assess calibration. Decision curve analysis was performed to quantify clinical utility. Duration of delirium, poor Richards–Campbell sleep questionnaire (RCSQ) score, advanced age, and sepsis were the most frequent and important candidates risk factors for PICS-CI. All ML models showed good performance (AUC range: 0.822–0.906). NN model had the highest AUC (0.906 [95% CI 0.857–0.955]), which was slightly higher than, but not significantly different from that of LR (0.898 [95% CI 0.847–0.949]) (*P* > 0.05, Delong test). Given the overfitting and complexity of some ML models, the LR model was then used to develop a web-based risk calculator to aid decision-making (https://model871010.shinyapps.io/dynnomapp/). In a low dimensional data, LR may yield as good performance as other complex ML models to predict cognitive impairment after ICU hospitalization.

## Introduction

Post-intensive care syndrome (PICS) is defined as development of new, or worsening of preexisting impairment in physical function, psychological disorder, or cognitive impairment that persists beyond ICU and hospital discharge^1^. Cognitive impairment related to PICS (PICS-CI) is one of the phenomena spanning from delirium to long-term cognitive impairment after discharge^2^, manifesting as memory loss, inattention, and/or executive dysfunction syndrome^3^. Neuroradiological and neuropathological studies in patients with PICS-CI have demonstrated diffuse brain damage, including global and local patterns of atrophy, as well as cortical and subcortical lesions, specifically in the corpus callosum, the hippocampus, and the basal ganglia^4^. PICS-CI is a common and severe complication of ICU admission. There is considerable variability in the reported incidence of PICS-CI after critical illness (19–78.2%)^5,6^. In a study, 23.9% patients showed persistence of cognitive deficit 6 years after discharge, and only 45.7% returned to the workforce^7^. PICS-CI after ICU admission can be greatly taxing to patients and their families^8^. The condition affects people of all ages^8^ and is associated with increased mortality^3^. Moreover, it adversely affects the activities of daily living and the quality of life, and the recovery process is typically protracted and incomplete over several years after discharge, and carries enormous societal cost^8^.

The need to detect PICS-CI is increasingly recognized. Several studies have investigated the risk factors for PICS, particularly preventable ones, but have yielded discrepant results. Some studies have identified delirium as a strong risk factor^6,9,10^. In a few studies, delirium exhibited a dose–response relationship with PICS. Longer duration of delirium showed an independent association with poor cognitive function^8^, and repeated delirium exposure was associated with a greater risk of developing cognitive deficit compared to single delirium episode^10^. Older age has not yet been conclusively determined as an independent risk factor, as no age-dependent increase in the prevalence of cognitive deficit was observed after ICU admission^8^; however, other studies support the hypothesis of a higher risk at older ages^11–13^. Some ICU-related parameters such as longer ICU length of stay and mechanical ventilation showed a significant positive association with the occurrence of cognitive impairment^13^. However, a meta-analysis confirmed that delirium is the sole risk factor for cognitive impairment^14^. A recent meta-analysis revealed a paucity of rigorous cognitive function prediction models, and identified the need for further studies in this field^15^.

Machine learning (ML) has been regarded as an indispensable tool for revealing complex questions in the field of medicine^16^. In critical care settings, ML applications have been leveraged to create predictive and prognostic models using supervised learning algorithms^17^. Complex ML algorithms (e.g., RF, NN, XGboost) have been claimed to present superior predictive discrimination compared to logistic regression technique^18–24^; nevertheless, the advantage of state of the art ML models over the conventional generalized linear models remains controversial^25–28^. For instance, two systematic reviews found no significant difference in performance between logistic regression and ML in clinical prediction modelling^25,28^.

To the best of our knowledge, no studies have developed and compared performance of multiple ML models for prognostic modeling in the context of PICS-CI. Therefore, the purpose of this study was to (1) identify significant risk factors for PICS-CI; (2) develop multiple ML models and compare these with traditional LR; (3) generate a visualization web-based calculator risk prediction model with the greatest possible accuracy.

## Materials and methods

### Study setting and design

We conducted a prospective observational study of ICU patients admitted between January and October 2019 at two tertiary hospitals in Fuzhou city, China, including general ICU, medical ICU, surgical ICU, cardiac ICU, and emergency ICU.

### Study population

Patients who qualified the following criteria were included in the study: (1) age > 18 years and < 80 years; (2) ICU length of stay > 48 h. Patients who qualified any of the following criteria were excluded: (1) nervous system diseases such as cerebral infarction, cerebral hemorrhage, meningitis, intracranial infection, craniocerebral injury, Parkinson’s, and brain tumor; (2) post-cardiopulmonary resuscitation or cardiac arrest; (3) pulmonary encephalopathy, hepatic encephalopathy, diabetic hyperosmolar coma, unexplained coma, and other diseases that may affect cognitive function; (4) patients with mental disorder, dementia/pre-dementia, or intellectual disability; (5) history of pesticide poisoning, drug poisoning, chronic alcohol abuse (consumption of alcohol for > 5 years, daily ethanol intake ≥ 80 g, calculation formula: ethanol volume (g) = consumed volume (mL) × ethanol concentration × 0.8); (6) Abuse of drugs such as hypnotics and anesthetics; (7) hearing impairment or visual impairment and inability to complete the cognitive assessment; (8) accepted pharmacological or non-pharmacological cognitive interventions; unwilling or unable to cooperate with assessment (e.g., due to tracheotomy).

### Procedure and data collection

After approval of the study protocol by the ethics institutional review board, which comply with Declaration of Helsinki, 3 researchers in each of the 5 ICUs underwent standardized training for data collection. The study nurse explained the purpose of the study to eligible patients and obtained written informed consent from those who agreed to participate, according to Declaration of Helsinki.

Two methods (on-spot assessment and review of medical records) were used for data collection. The collected data were classified into 5 categories (a total of 35 variables), including demographics, disease-related features, treatment-related features, laboratory test features, and sleep quality. Sleep quality was assessed daily during the period of stay in the ICU. Screening for cognitive impairment was conducted by researcher after patient was discharged and returned to the general ward. Other factors were measured and recorded in the electronic medical record (EMR) by medical staff, and we extracted and confirmed the information with the patient. The data collection process is shown as follows.

#### Demographics

Demographic information was obtained from the EMR including age, sex (male or female), educational level (illiterate, primary school, junior high school, high school or secondary vocational school, college school or above), occupation (physical labor/nonphysical labor), body mass index, and history of alcohol consumption.

#### Disease-related features

The disease-related parameters were extracted from the ICU discharge diagnosis from EMR, including diagnosis (sepsis, hypertension, diabetes, chronic cardiorespiratory disease, chronic renal disease, respiratory failure, etc.). We classified it as acute disease, acute exacerbation of chronic disease, cardiac surgery, and non-cardiac surgery. We also calculated the Charlson Comorbidity Index (CCI) to evaluate the severity of complications, and the Acute Physiology and Chronic Health Status Score (Acute Physiology and Chronic Health Evaluation II, APACHE II).

#### Treatment-related features

The treatment-related factors were summarized after discharge from the ICU, which were daily assessed and recorded in the EMR by medical staff, including duration of mechanical ventilation, cumulative doses of sedatives (propofol and midazolam), and cumulative doses of analgesics (sufentanil and remifentanil), duration of ICU stay, delirium, and duration of delirium.

Delirium was assessed using the Confusion Assessment Method for Intensive Care Unit questionnaire (CAM-ICU)^29^ during ICU hospitalization. Delirium assessments were conducted twice daily (before 10 AM and after 8 PM) by the charge nurse. Before each delirium assessment, the Richmond Agitation and Sedation Scale (RASS)^3^ was used to assess the level of consciousness; patients with scores of − 4 or − 5 were not tested for delirium; delirium was assessed only when RASS score was below − 3. CAM-ICU delirium was deemed positive when there was an acute change in mental status from baseline with difficulty in focusing attention in combination with altered level of consciousness or disorganized thinking. Duration of delirium referred to the number of days on which delirium was observed.

#### Laboratory test features

The laboratory data were derived from EMR after ICU discharge, including oxygenation index PaO_2_/FiO_2_ (minimum), procalcitonin (maximum), glucose (minimum and maximum), WBC count (minimum and maximum), and lactate (maximum).

#### Sleep quality

Nocturnal sleep quality was evaluated daily by charge nurse at 9 AM using Richards-Campbell Sleep Questionnaire (RCSQ)^30^ that comprises of five items: sleep depth, sleep latency, awakenings, returning to sleep, and sleep quality. Each item was rated on a 100-mm visual analog scale. Participants with a score of 1–25 were considered to have poor sleep quality; score of 26–50 indicated poor sleep; score of 51–75 indicated good sleep; and score of 76–100 indicated very good sleep.

#### Outcome measurement

Cognitive status was measured using Montreal Cognitive Assessment (MoCA) scores by researcher within 7 days after the transfer of the patient out of the ICU. MoCA is a brief but reliable test for evaluation of visuospatial/executive visuospatial/executive function, naming, memory, attention, language, abstraction, delayed recall and orientation, with a total score of 0–30 (the higher the score, the better is the function)^31^. Changsha version of MoCA-CS is a Chinese version of the original (English) version of MoCA that is adapted to Chinese culture, language, and demographic characteristics^31^. Use of 25/26 as the optimal cutoff value (≥ 26 points: normal cognitive function; ≤ 25 points: cognitive impairment) was associated with sensitivity of 0.96 and specificity of 0.83 in Chinese older people. MoCA has been recommended for screening of PICS-related cognitive impairment by the Society of Critical Care Medicine consensus^32^.

### Statistical analysis

All statistical analyses were conducted using R version 4.1.2 (R Foundation for Statistical Computing, Vienna, Austria). Categorical variables are presented as frequency (percentage). Normally distributed continuous variables are presented as mean ± standard deviation and non-normally distributed continuous variables are presented as median (interquartile range). Comparisons between PICS and non-PICS group were performed using the *t* test, Mann–Whitney U-test, Fisher’s exact test, or Chi squared test, as appropriate. *P* values < 0.05 were considered indicative of statistical significance.

#### Data preparation

**Figure 1:**
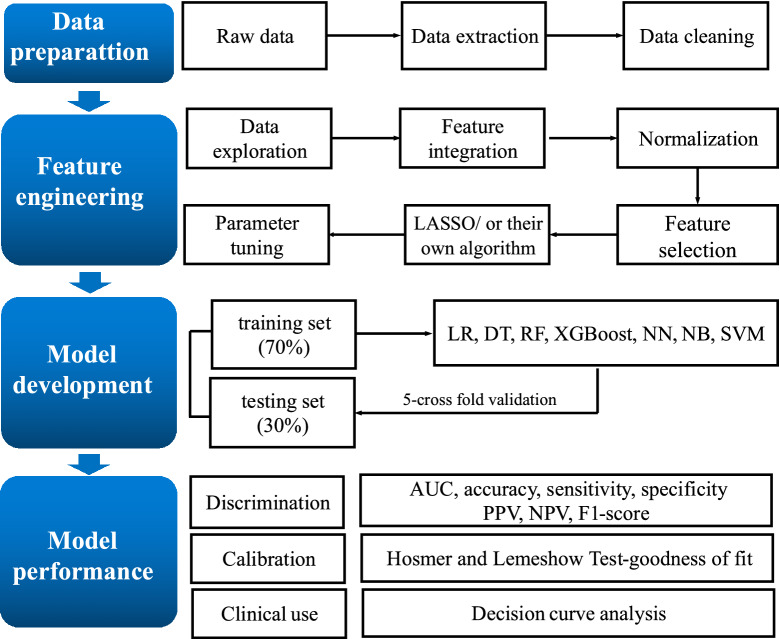
Flow chart of the probability analysis. *LR* logistic regression, *DT* decision tree, *RF* random forest, *NN* neural network, *NB* naïve bayes, *SVM* support vector machine, *AUC* area under the receiver operating characteristic curve, *NPV* negative predictive value, *PPV* positive predictive value.

The flow chart of the probability analysis is shown in Fig. 1. We adopted the imputation and discretization methods to clean data and to deal with noise, missing values, and outliers. To ensure the facticity and reliability of the prediction model, we discarded one variable with 23.91% missing data, namely the maximum value of CRP; the missing values for 5 other variables were < 10%, therefore, multiple imputation using the mice package was performed.

#### Feature selection

Feature selection is used to improve model performance and retrieve more clinically applicable compact models. We normalized the data and target values locked at 0–1. A total of 35 candidate features describing PICS-CI risk were collected in this study. We used least absolute shrinkage and selection operator (LASSO) regression as a variable selection mechanism for the algorithms that were unable to select features for themselves, such as XGBoost, neural network (NN), naïve Bayes (NB), and support vector machine (SVM); however, for models such as logistic regression (LR), decision tree (DT), random forest (RF), we used their own algorithms for feature selection.

#### Model development

The dataset was randomly split into two sets: the training set (70% participants) and the testing set (30% participants). Seven ensemble ML algorithms were employed to predict the probability of PICS-CI. These seven state-of-the-art algorithms were LR, DT, RF, XGBoost, neural network (NN), naïve Bayes (NB), and support vector machine (SVM). To select the optimal hyperparameters, the framework of the *caret* package was used. The conventional LR model was selected as a benchmark to compare with ML techniques. The LR model was conducted using significant variables identified by backward stepwise analysis with Chi-squared test. Then, we chose an entry probability of < 0.05 by the stepwise selection method, and the *glmnet* function from the *glmnet* package was used.

#### Model validation

To cope with the overfitting and inherent instability, a fivefold cross-validation procedure was applied in the training set. Subsequently, the performance of the final model was assessed, with the optimal cutoff identified by the maximum Youden index, both on the training set and the testing set.

#### Model evaluation

The performance was assessed in three domains. First, discrimination was quantified using the area under the receiver operating curve using the ci.auc function of the *pROC* package. AUC > 0.5 indicated better predictive values; the closer the AUC value was to 1, the better the model performance. The differences between two ROC curves were assessed using the DeLong’s test. More extensive metrics were generated for each model including accuracy, sensitivity, specificity, positive predictive value (PPV), negative predictive value (NPV), and F1 score. Second, calibration curves were plotted to assess the calibration of the PICS-CI, accompanied with the Hosmer–Lemeshow test (a significant test statistic implies that the model does not calibrate perfectly), in order to examine the concordance between the calculated (using model) and observed probabilities of insufficient response. Third, decision curve analysis was performed to assess the benefits of clinical use. After comparing the seven models, the most accurate model was then used to construct a web-based risk calculator by *shiny* package in the R language.

### Ethics statement

The study protocol was approved by the Fujian Provincial Hospital Institutional Review Board ([No:2016-07-001), and the purpose of the study was explained to eligible patients and obtained written informed consent from those who agreed to participate.

## Results

### Patient characteristics

**Figure 2:**
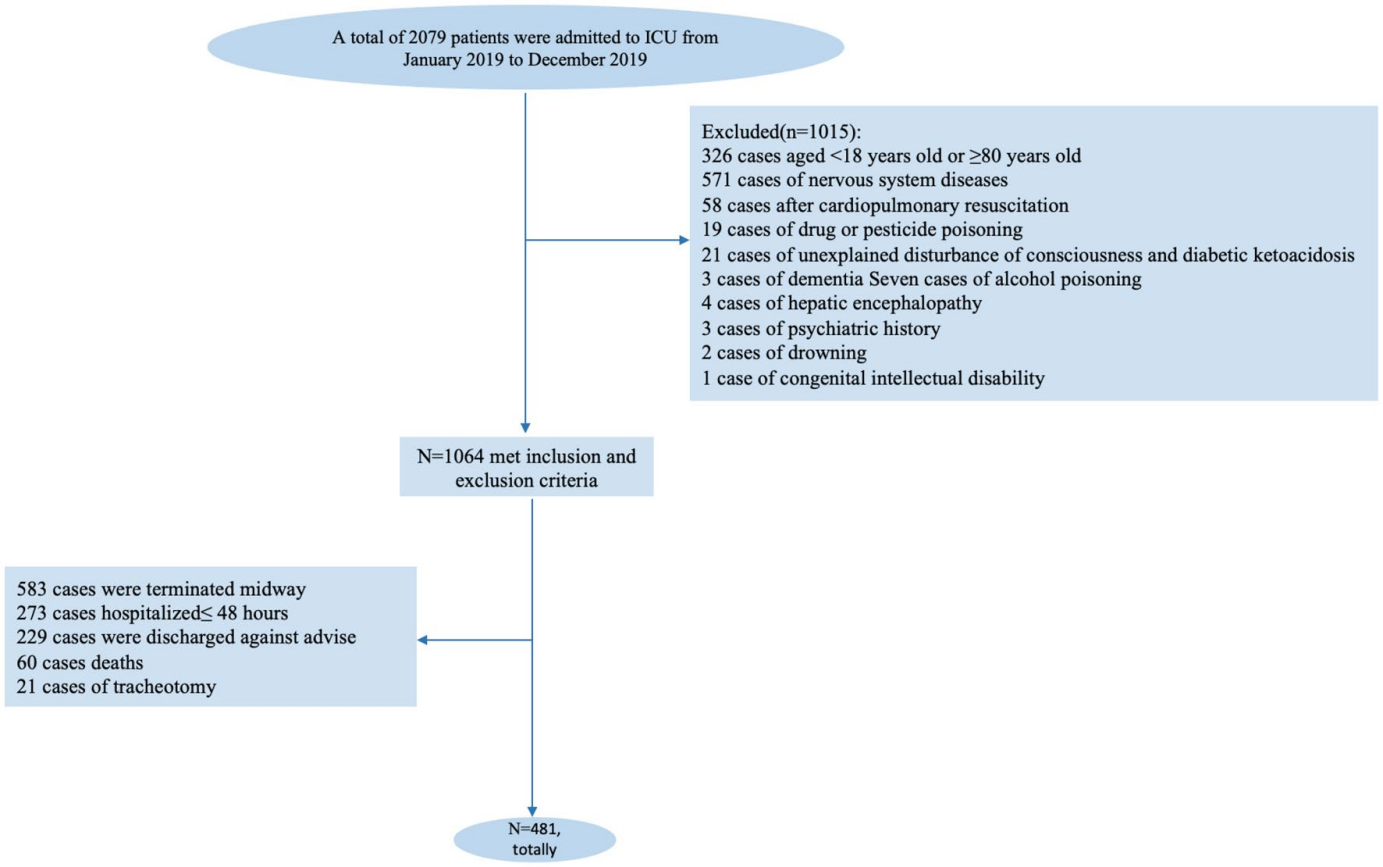
Flowchart of participants.

Among the 2079 patients admitted to ICUs at the two tertiary hospitals within the study reference period, 1064 patients qualified the study-selection criteria. Of these 583 patients were excluded due to various reasons (hospitalization < 48 h, discharge against medical advice, death, or inability to communicate due to tracheotomy), as shown in Fig. 2. Thus, a total of 481 patients were finally included; of these, 230 were diagnosed as PICS-CI while 251 had normal cognitive function. The patient characteristics are summarized in Table 1.

**Table 1:** Characteristics of the study population.

Features		PICS-CI		*χ*^2^/*Z*	*P* value
		No n = 251	Yes n = 230		
Continuous variable, [N (%)]					
Sex	Male	170 (67.7)	153 (66.5)	0.079	0.778
Marital status	Married	230 (91.6)	215 (93.5)	2.432	0.272
	Widowed	4 (1.6)	6 (2.6)		
	Others	17 (6.8)	9 (3.9)		
Education	Less than 6 years	80 (31.9)	111 (48.3)	14.844	0.001
	6 to 12 years	149 (59.3)	109 (47.4)		
	More than 12 years	22 (8.8)	10 (4.3)		
Smoking		63 (25.1)	51 (77.8)	0.568	0.451
Sepsis		20 (8.0)	39 (17.0)	9.01	0.003
Diagnosis	Infectious disease	20 (8.0)	29 (12.6)	8.25	0.220
	Respiratory diseases	43 (17.1)	33 (14.3)		
	Kidney disease	7 (2.8)	5 (2.2)		
	Traumatic disease	20 (8.0)	13 (5.7)		
	Digestive system diseases	55 (21.9)	43 (18.7)		
	Cardiovascular diseases	89 (35.5)	98 (42.6)		
	Others	17 (6.7)	9 (3.9)		
T ≥ 38 °C		111 (44.2)	122 (53.0)	3.739	0.053
Categorical variable, [M(IQR)]					
Age, year		56.0 (22.0)	63.0 (18.0)	− 5.11	0.00
Height		168.0 (13.0)	167.0 (11.3)	− 1.83	0.07
Weight		64.0 (13.0)	63.0 (14.0)	− 0.79	0.43
APACHEII		14.0 (8.0)	16.0 (10.0)	− 2.65	0.01
SOFA		5.0 (5.0)	6.0 (5.0)	− 2.73	0.01
CCI		2.0 (3.0)	2.0 (2.0)	− 1.33	0.18
ICU length of stay, day		7.0 (8.0)	8.0 (10.3)	− 2.60	0.01
Duration of mechanical ventilation, hours		0.0 (18.0)	45.0 (120.0)	− 7.46	0.00
Sedative					
Propofol dose, g		0.0 (1.0)	1.4 (4.6)	− 7.25	0.00
Midazolam dose, mg		0.0 (20.0)	20.00 (260.0)	− 5.94	0.00
Analgesic					
Remifentanil dose, mg		0.0 (2.0)	1.0 (9.0)	− 4.36	0.00
Sufentanil dose, mg		0.0 (300.0)	200.0 (600.0)	− 4.69	0.00
Norepinephrine		2.0 (30.0)	8.0 (66.0)	− 4.12	0.00
Dopareine		0.0 (20.0)	0.0 (20.0)	− 3.36	0.00
Dobutamine		0.0 (0.0)	0.0 (0.0)	− 4.18	0.00
Duration of delirium, days		0.0 (0.0)	3.0 (6.0)	− 13.77	0.00
0		254 (69.4)	112 (30.6)	− 7.48	0.00
< 3		5 (21.7)	18 (78.3)		
3–7		4 (6.8)	55 (93.2)		
> 7		3 (9.1)	30 (90.9)		
RCSQ-sleep depth		65.0 (20.0)	55.0 (20.0)	− 7.48	0.00
RCSQ-sleep latency		66.0 (20.0)	55.0 (20.0)	− 7.36	0.00
RCSQ-awakenings		70.0 (15.0)	60.0 (15.0)	− 6.75	0.00
RCSQ-returning to sleep		70.0 (15.0)	60.0 (15.0)	− 8.71	0.00
RCSQ-sleep quality		70.0 (15.0)	60.0 (15.0)	− 8.19	0.00
RCSQ-average		69.0 (16.0)	56.1 (13.3)	− 8.67	0.00
RCSQ-noise		60.0 (15.0)	55.0 (10.0)	− 6.58	0.00
Glucose, mmol					
Minimum value		5.2 (1.7)	5.4 (1.7)	− 1.33	0.18
Maximum value		14.1 (7.3)	15.9 (7.9)	− 3.76	0.00
PCT maximum value, ng/ml		1.8 (8.7)	4.3 (15.7)	− 3.48	0.00
WBC maximum value, count		15.3 (8.0)	16.9 (8.2)	− 2.63	0.01
Lac, mmol		2.9 (5.8)	3.9 (8.5)	− 2.50	0.01
PaO_2_/FiO_2_		170.0 (113.0)	152.2 (118.0)	− 3.01	0.00

### Important variables selected for predicting PICS-CI

**Figure 3:**
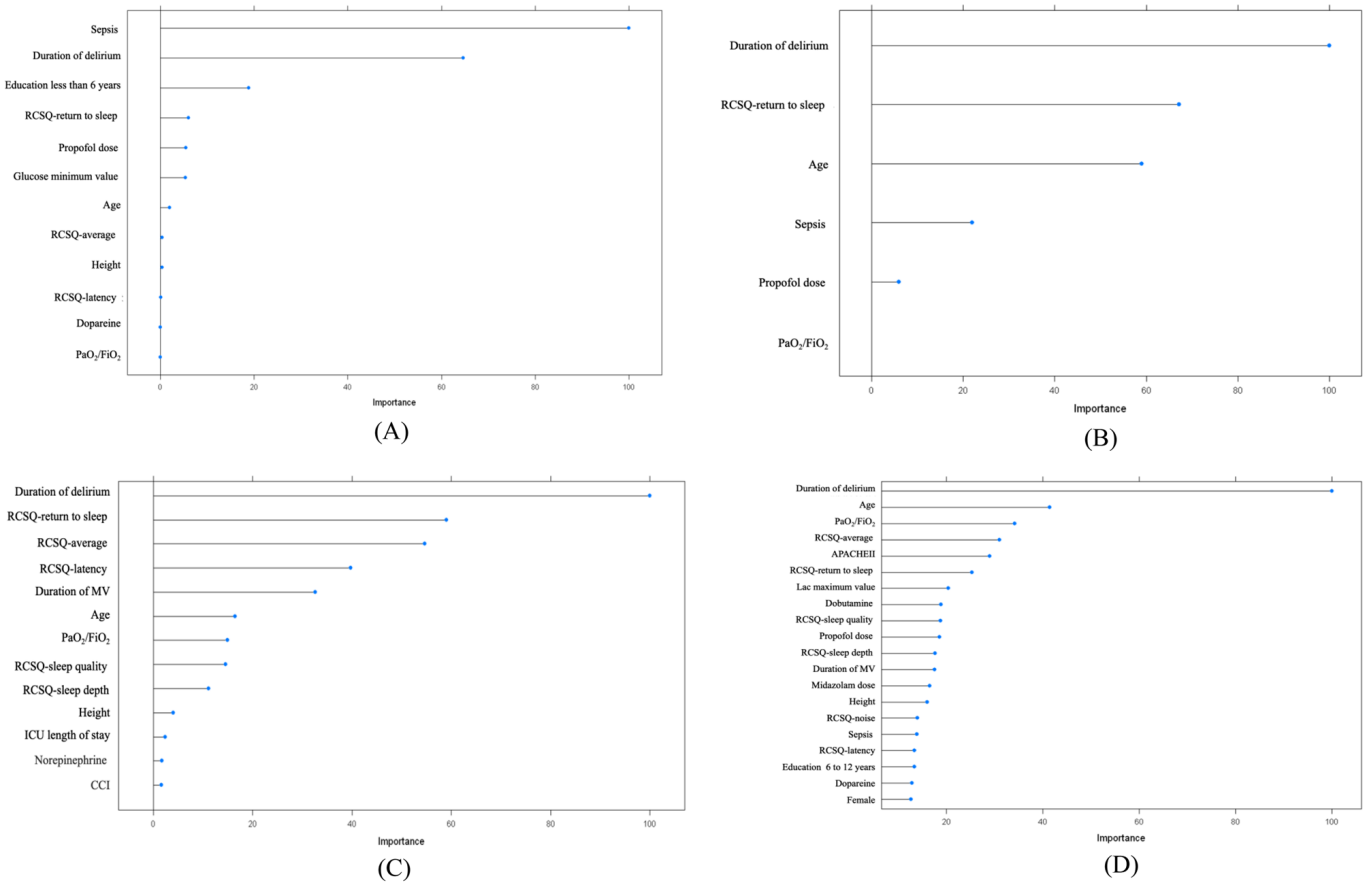
Importance of the candidate features. (**A**) Feature selection by LASSO, (**B**) feature selection by logistic regression, (**C**) feature selection by decision tree, (**D**) feature selection by random forest.

Figure 3 depicts the four kinds of important features that were generated by LASSO, multivariate LR, DT, and RF, respectively. XGBoost, NN, NB, and SVM models were developed based on the different combination of the features that were selected by LASSO, while the LR, DT, and RF models were developed by candidate features selected by their own algorithms. Features that were important for all algorithms were duration of delirium, RCSQ, age, and sepsis.

### Discrimination of ML models for predicting PICS-CI

**Figure 4:**
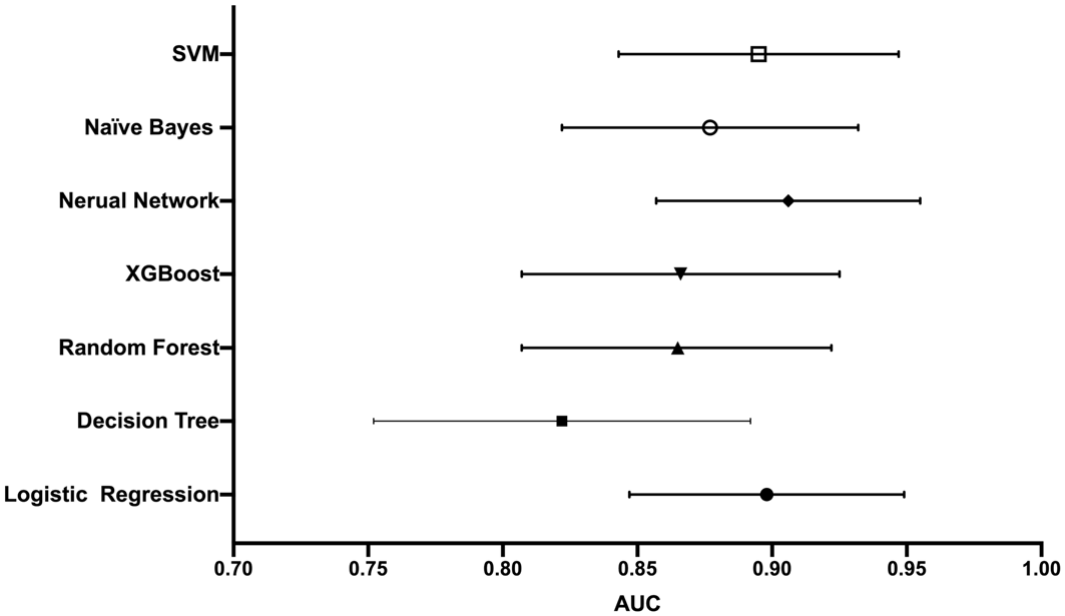
AUC of machine learning models predicting PICS-CI of testing set.

In the testing set, ML models showed similar discrimination: NN (AUC: 0.906 [95% CI 0.857–0.955]), LR (AUC: 0.898 [95% CI 0.847–0.949]), SVM (AUC: 0.895 [95% CI 0.843–0.947)], NB (AUC: 0.877 [95% CI 0.822–0.932]), XGBoost (AUC: 0.866 [95% CI 0.807–0.925]), RF (AUC: 0.865 [95% CI 0.807–0.922]), DT (AUC: 0.822 [95% CI 0.752–0.892]), shown in Fig. 4. The Delong test showed that only DT model had significant difference (significantly lower AUC) when compared with LR technique (*P* < 0.05), whereas, other ML models showed no significant difference in the discrimination ability (*P* > 0.05). In terms of confusion matrix, LR showed the highest sensitivity of 0.899, but lowest specificity of 0.750; other ML models produced opposite results, i.e., poor sensitivity (0.594–0.783) but excellent specificity (0.895–1.00), shown in Table 2.

**Table 2:** The performance of machine learning models.

	Type	Cut off	Specificity	Sensitivity	Accuracy	NPV	PPV	Precision	Recall	F1 score	AUC (95%CI)
Training set	LR	0.453	0.880	0.752	0.819	0.794	0.852	0.852	0.752	0.799	0.896 (0.863–0.928)
	DT	0.550	0.926	0.758	0.845	0.806	0.904	0.904	0.758	0.824	0.878 (0.840–0.915)
	RF	0.391	0.903	0.969	0.936	0.969	0.902	0.902	0.969	0.934	0.979 (0.968–0.991)
	XGBoost	0.403	0.874	0.857	0.866	0.869	0.863	0.863	0.857	0.860	0.935 (0.910–0.960)
	NN	0.510	0.920	0.752	0.839	0.801	0.896	0.896	0.752	0.818	0.905 (0.874–0.936)
	NB	0.037	0.811	0.776	0.795	0.798	0.791	0.791	0.776	0.784	0.862 (0.823–0.901)
	SVM	0.370	0.817	0.820	0.819	0.831	0.805	0.805	0.820	0.812	0.901 (0.869–0.933)
Testing set	LR	0.369	0.750	0.899	0.821	0.891	0.765	0.765	0.899	0.827	0.898 (0.847–0.949)
	DT	0.926	1.000	0.594	0.807	0.731	1.000	1.000	0.594	0.746	0.822 (0.752–0.892)*
	RF	0.545	0.934	0.638	0.793	0.740	0.898	0.898	0.638	0.746	0.865 (0.807–0.922)
	XGBoost	0.587	0.961	0.638	0.807	0.745	0.936	0.936	0.638	0.759	0.866 (0.807–0.925)
	NN	0.551	0.895	0.783	0.841	0.819	0.871	0.871	0.783	0.824	0.906 (0.857–0.955)
	NB	0.307	0.934	0.681	0.814	0.763	0.904	0.904	0.681	0.777	0.877 (0.822–0.932)
	SVM	0.480	0.908	0.754	0.835	0.802	0.881	0.881	0.754	0.813	0.895 (0.843–0.947)

*LR* logistic regression, *DT* decision tree, *RF* random forest, *NN* neural network, *NB* naïve bayes, *SVM* support vector machines, *NPV* negative predictive value, *PPV* positive predictive value.

*The AUC of DT model was significantly different from that of LR algorithm (Delong test), while others not.

### Calibration of ML models for predicting PICS-CI

**Figure 5:**
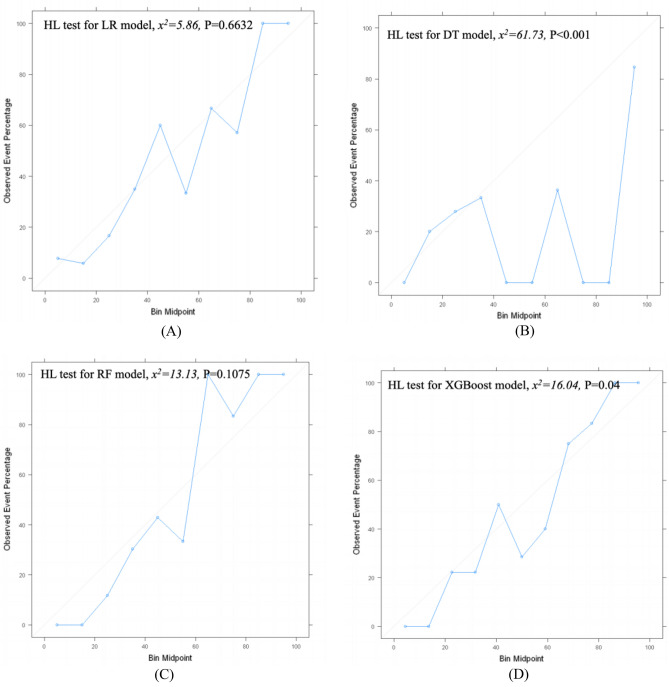

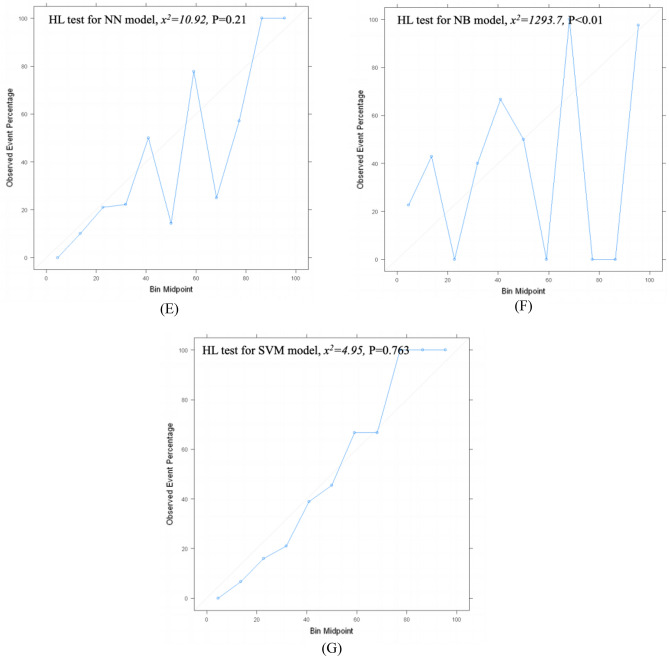
Calibration curve and HL-test result of machine learning models of testing set. (**A**) for logistic regression, (**B**) for decision tree, (**C**) for random forest, (**D**) for XGBoost, (**E**) for neural network, (**F**) for naïve bayes, (**G**) for support vector machine.

Hosmer and Lemeshow Test-goodness of fit demonstrated that LR, RF, NN, and SVM models have good agreement between the predicted probability and observed incidence of PICS-CI (*P* > 0.05); nevertheless, unacceptable calibration was observed for DT, XBGoost, and NB models (*P* < 0.05) (Fig. 5).

### Clinical use of ML models for predicting PICS-CI

**Figure 6:**
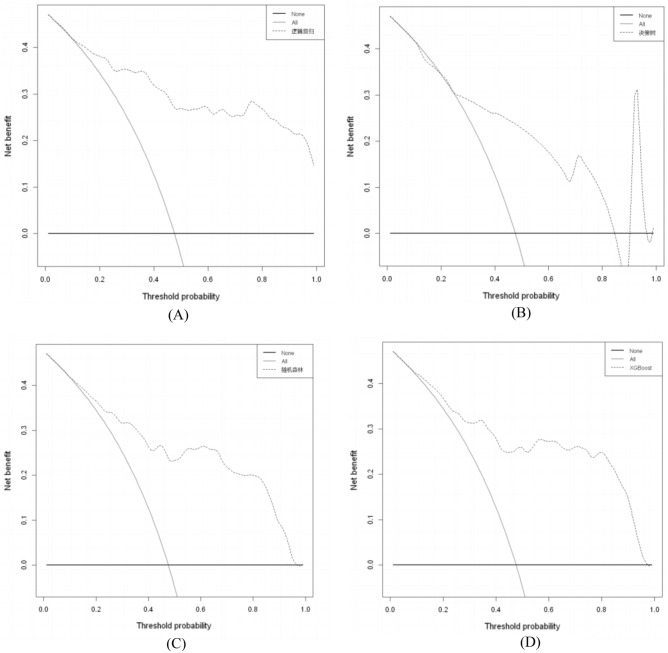

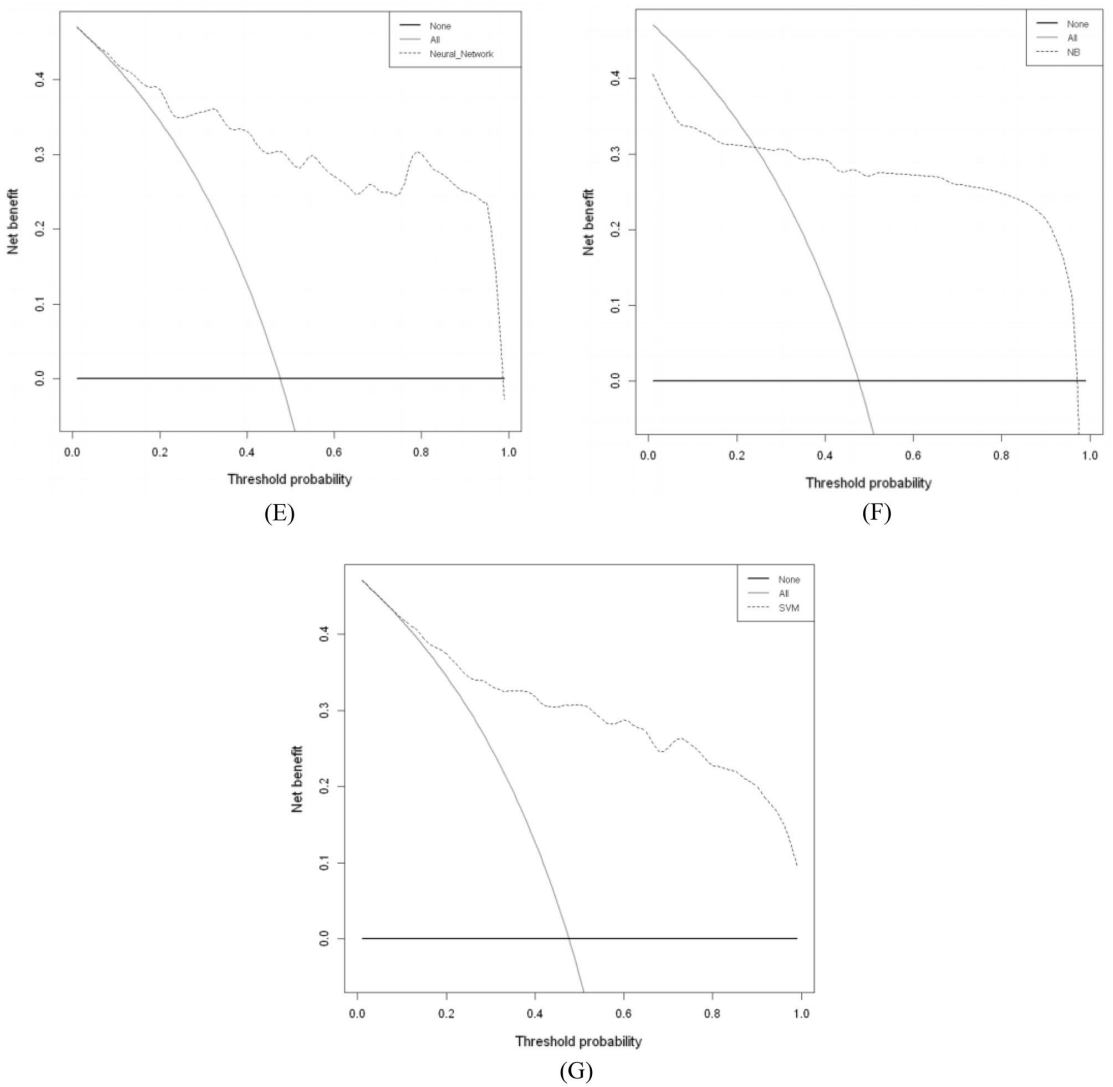
Decision curve analysis of machine learning models of testing set. The y-axis measures the net benefit. DCA shows the clinical usefulness of the machine learning models, according to a continuum of potential thresholds for PICS-CI risk (x-axis) and the net benefit of using the prediction model to stratify patients according to risk (y-axis). Transverse line represents the assumption that no patients have PICS-CI; oblique line represents the assumption that all patients have PICS-CI; dotted line represents the PICS-CI model). (**A**) For logistic regression, (**B**) for decision tree, (**C**) for random forest, (**D**) for XGBoost, (**E**) for neural network, (**F**) for naïve bayes, (**G**) for support vector machine.

The decision curve analysis for predicting PICS-CI is presented in Fig. 6. Most of the ML models of LR, RF, XGBoost, NN, and SVM showed a higher net benefit compared to both treat all (grey line) and treat none (black line) with a wide range of reasonable thresholds probability; however, the benefit of DT and NB models was comparatively lower, as depicted in Fig. 6.

### Web-based risk calculator of LR technique

**Figure 7:**
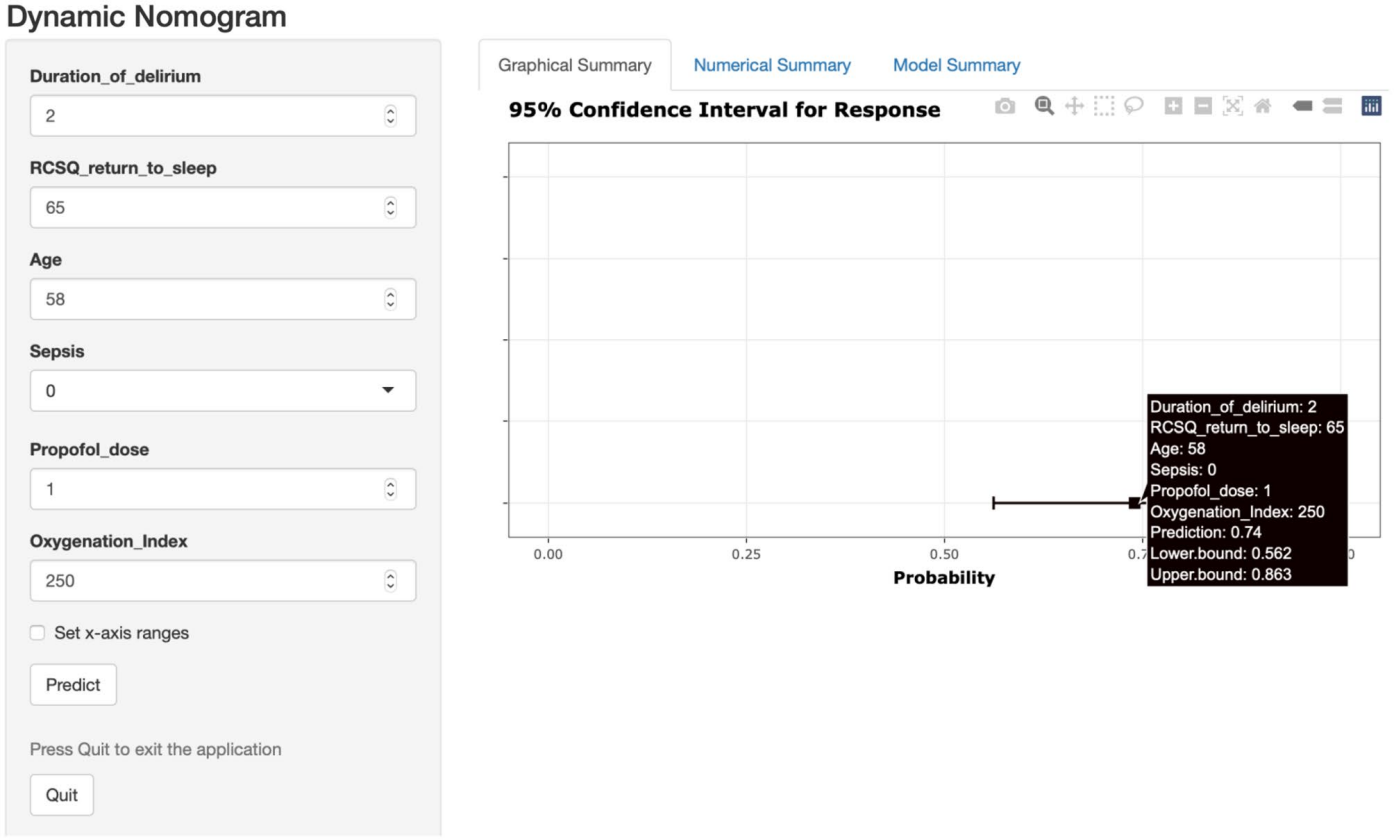
Web-based risk calculator of PICS-CI (Example of a 58-year-old critically ill patient complicated with delirium for 2 days and having fair sleep quality (RCSQ-returning to sleep = 65), no sepsis, use propofol, and PO_2_/FiO_2_ value of 250 in daily assessment; this patient would have a 74% risk of PICS-CI).

Considering the performance and complexity of most ML models, we regarded LR as an accurate and pragmatic model, and given the number of variables included in the final LR model, a simple web-based risk calculator (rather than a score chart or nomogram) was developed and housed at the website: https://model871010.shinyapps.io/dynnomapp/. The website offers a visual representation of the predicted probability of PICS-CI and its 95% prediction interval based on the variables’ input (Fig. 7).

## Discussion

In the present study, we explored and compared the applicability of seven ML methods to predict the risk of PICS-CI in a low dimensional data. LR models exhibited accuracy and similar discrimination, good calibration and clinical utility among the seven ML models. Given the risk of overfitting and the lack of interpretability of some ML models, a simplified web risk calculator based on LR was proposed, which is convenient to implement in routine practice. Therefore, traditional regression models should continue to play a key role in disease risk prediction if and when low-dimensional data are used.

To the best of our knowledge, this was the first study to develop and compare ML models to predict the risk of PICS-CI. Our analysis indicated that the performance of complex ML models is not consistently better than traditional LR models in predicting PICS-CI events. Indeed, some models had lower performance, such as the DT model which significantly underperformed the LR model (*P* < 0.05 for Delong test). One potential reason why ML classification algorithms did not outperform traditional LR models in our study may be that PICS-CI is an easily predictable behavior, and all the models were performing similarly well (AUC range: 0.822–0.906). It is also possible that we have collected sensitive variables predicting PICS-CI, and these variable domains can improve the prediction of appropriate PICS-CI. In fact, the added value of large datasets and the increase in algorithmic complexity may be negligible if the relevant features are not included^33^.

We identified a potentially modifiable or unchangeable risk factor for post-ICU cognitive impairment. We found that duration of delirium, poor RCSQ, advanced age, and sepsis were the most frequent and important candidates. In the present study, for each additional day of delirium, the risk of PICS-CI was increased by 2.64 times. Several prior studies have yielded consistent results. In a single-center prospective study of 409 ICU patients, compared with no delirium experience, the occurrence of PICS-CI increased by 36.849 times with a wide 95% CI when the duration of delirium was greater than 8 days^34^. A meta-analysis even suggested that delirium is likely to be the sole risk factor for PICS-CI^14^. We used RCSQ to measure patient perception of sleep quality, including sleep depth, sleep latency, awakenings, returning to sleep, and sleep quality^35^. We found that patient self-reported sleep quality was also associated with PICS-CI. One possibility is that sleep deprivation and delirium share many common symptoms; both these factors are likely to have a bidirectional effect and may create a potential vicious cycle in critically-ill patients^36^. Sleep fragmentation has also been shown to be associated with worse cognitive performance in hospital at 7 days of ICU discharge^37^. However, Yao et al. found that the RCSQ is not effective in predicting PICS-CI; this may be due to the fact that they just assessed sleep abnormalities occurring in critically-ill patients within 3 days after ICU discharge^34^. In our study, advanced age was found to be a risk factor for PICS-CI. This result should be interpreted with caution, because cognitive function is an age-dependent syndrome. In addition, we did not measure the cognitive function prior to ICU admission, so it is difficult to determine that it was acquired after critical illness. Sepsis-related encephalopathy is characterized by pathological behavior ranging from delirium to coma, and may lead to long-term cognitive impairment^38^, affecting up to 50% of patients during the course of sepsis^39^. The pathogenesis is due to a dysregulated host response and the absence of direct central nervous system infection, resulting in residual diffuse brain dysfunction^40^.

Second, the inherent flexibility and scope for automation make ML well suited to handling complex high-dimensional data (i.e., with many variables or features, perhaps more than 50 predictors) that would be challenging for conventional approaches^26,33^. The flexibility of ML algorithms is manifested in their ability to capture complex nonlinear and interactive effects^41^, which may be a common feature of high-dimensional data. In the present study, we used a limited set of clinical predictors (n = 35) which may have contributed to the comparable performance of ML and traditional method. In a study using large data set (n = 11,022) with low dimensional data (only 11 variables), ML methods were not found to improve prognostic prediction as well^26^. As suggested by previous studies, when the clinical data is low-dimensional data (often with limited data set, a small number of clinical predictors, few nonlinear and interactive effects), LR is an easy-to-use and appropriate model for disease risk prediction^28^. The ability of ML to process high-dimensional data should not distract from the often-greater benefits of traditional LR.

Finally, despite the good discriminatory power of some ML models, for instance, DT (AUC: 0.822 [95% CI 0.752–0.892]), XGBoost (AUC: 0.866 [95% CI 0.807–0.925]), and NB (AUC: 0.877 [95% CI 0.822–0.932]), these models may not end up being used, because these clearly underperformed in terms of calibration (*P* < 0.05). This indicates that overfitting is a common problem arising in small data sets^42^. In contrast, DT, XGBoost, and NB methods have exhibited better performance in other studies^43–45^, which however did not assess calibration. A recent systematic review^28^ did not find an incremental value of flexible ML techniques over traditional statistical methods in relatively small data sets (median sample size: 1250); moreover, calibration was not addressed in 79% of ML studies. Calibration evaluation is a critical step before implementing predictive models in clinical practice. Moreover, reporting guidelines for building predictive models using ML methods recommend reporting calibration^46^.

Given the lack of interpretability of some ML models, we therefore presented a web-based risk calculator based on LR model, which could allow for a more precise predicted probability compared to a points-based scoring system. For example, a 72-year-old critically ill patient complicated with delirium for 3 days and having fair sleep quality (RCSQ-returning to sleep = 60), no sepsis, no propofol, and minimum value of PO_2_/FiO_2_ in daily assessment, would have an 87% risk of PICS-CI. However, in another patient with similar characteristics, except having no delirium, the observed probability would reduce to 49%.

Some limitations of our study should be acknowledged. First, we considered only a limited number of variables for predicting PICS-CI. We restricted our analyses to predictive modeling with known or potential risk factors. This may limit the generalizability of our conclusion to data sets with more variables. Second, though the several ML models demonstrated perfect performance in internal validity, further research should use a prospective external validation to check for web-based risk calculator for routine clinical use. Third, the present study was unable to measure cognitive function prior to ICU admission which may have possibly contributed to overestimation of the incidence of ICU-acquired cognitive impairment. Of note, we excluded patients aged ≥ 80 years and those with nervous system disease as in these patients, the cognitive impairment may be due to physiology or the disease itself rather than ICU stay. Finally, we only investigated short-term cognitive impairment during hospitalization and did not conduct a long-term follow-up after discharge.

## Conclusion

Our results indicate that the risk of early cognitive impairment post ICU hospitalization can be parsimoniously assessed by several ML prediction models. Indeed, in a low-dimensional and simple limited data setting, complex ML algorithms performed no better than traditional LR model for prediction of PICS-CI. We hope that LR model with its accompanying web-based risk calculator will facilitate prevention and early recognition of PICS-CI.

## Data Availability

The data that support the findings of this study are available from the corresponding author upon reasonable request.

## Abbreviations

AUC: Area under the receiver operating characteristic curve
CAM-ICU: Confusion assessment method for intensive care unit questionnaire
CCI: Charlson comorbidity index
DT: Decision tree
RF: Random forest
LR: Logistic regression
ML: Machine learning
MoCA: Montreal cognitive assessment
NN: Neural network
NB: Naïve bayes
NPV: Negative predictive value
PICS-CI: Cognitive impairment related to post intensive care syndrome
PPV: Positive predictive value
RCSQ: Richards–Campbell sleep questionnaire
SVM: Support vector machine
WBC: White blood cell
